# Biology

**Published:** 1877-12

**Authors:** 


*Biology, with Preludes on Current Events.* By JOSEPH COOK, Boston: James R. Osgood & Co., 1877. 12 mo. pp. 325. Price, $1.50.

Thirteen Lectures comprising the forty-sixth to the fifty-eighth lectures in the Boston Monday Lectureship are here presented. The latest thoughts of the greatest minds of Europe and America, upon the wide domain of Biology are given to the public in a concise and pleasing style.

The part of the work comprising the two lectures upon Living Tissues and Life or Mechanism, is an epitome of our positive knowledge in biological science. The thirty-four points on pages 124-126, should be imprinted upon the memory of every thoughtful man. These three pages alone are worth much more than the price of the book. Our advice is to buy and carefully read it. C.

---

*A Family Newspaper.*

*The Family Newspaper* should have attractive reading and information for the various members of a household. Some portion of the paper should be devoted, every week, to religious and moral inprovement, to current secular news, to agriculture, commerce, markets, finance, to general literature, &c., with a special department for the young. Above all, the *Family Newspaper* should be perfectly *pure*, and free from any contaminating influences in its reading matter or in its advertisements. Too much attention cannot be paid to this feature, when the press is flooding the country with so much that is vile and pernicious. To crown all, the *Family Newspaper* should be untrammelled by any affiliation with sect or party, and should be free to give all the good news from and about all the world. If such a *Family Newspaper* can be had for *one cent a day*, it should be taken by every family in the land.

Such a *Family Newspaper*, in every respect, we find in the NEW YORK OBSERVER, now commencing its fifty-sixth volume. Progressive, comprehensive, sound, reliable, pure, it is just what is needed in your household. Send $3.15 for a year to THE NEW YORK OBSERVER, 37 Park Row, New York. Sample copies are sent free.

